# Connection adaption for control of networked mobile chaotic agents

**DOI:** 10.1038/s41598-017-16235-2

**Published:** 2017-11-22

**Authors:** Jie Zhou, Yong Zou, Shuguang Guan, Zonghua Liu, Gaoxi Xiao, S. Boccaletti

**Affiliations:** 1 School of Physics and Materials Science, East China Normal University, Shanghai, 200241 China; 2 School of Electrical and Electronic Engineering, Nanyang Technological University, Nanyang, 639798 Singapore; 3 Complexity Institute, Nanyang Technological University, Nanyang, 639798 Singapore; 4 CNR-Institute of Complex Systems, Via Madonna del Piano, 10, 50019 Sesto Fiorentino, Florence, Italy; 5 The Embassy of Italy in Tel Aviv, 25 Hamered street, 68125 Tel Aviv, Israel

## Abstract

In this paper, we propose a strategy for the control of mobile chaotic oscillators by adaptively rewiring connections between nearby agents with local information. In contrast to the dominant adaptive control schemes where coupling strength is adjusted continuously according to the states of the oscillators, our method does not request adaption of coupling strength. As the resulting interaction structure generated by this proposed strategy is strongly related to unidirectional chains, by investigating synchronization property of unidirectional chains, we reveal that there exists a certain coupling range in which the agents could be controlled regardless of the length of the chain. This feature enables the adaptive strategy to control the mobile oscillators regardless of their moving speed. Compared with existing adaptive control strategies for networked mobile agents, our proposed strategy is simpler for implementation where the resulting interaction networks are kept unweighted at all time.

## Introduction

Synchronization is a ubiquitous collective behavior, widely found in biological, physical, and social systems^1–6^. An issue of particular relevance is the control of networked systems, so as to target them towards specific, collective, and desired functionings. When the topology of connections among the units of a network is fixed, various strategies can be adopted, such as: *i*) regulating the strength of the coupling between the graph’s components^7–13^, *ii*) applying a time delay^14,15^, *iii*) changing adaptively the nodes’ interaction^16^, *iv*) pinning specific sequences of network’s components^17–19^. With the rapid development of technology in mobile devices, synchronization and control of mobile agents becomes, however, an issue of primary importance^20–22^. Conditions for synchronization of networks with time-varying structure under various circumstances have been studied. Such conditions include that the time-varying structures are always small world^23,24^, oscillators perform random walk on networked infrastructure^25–27^, oscillators could move on a one-dimensional ring^28^, oscillators are allowed to jump randomly in the space^29^, etc. A good study of the condition of stable synchronization for fast-switching networks, where the network structure changes much faster than the unit dynamics, can be found in ref.^30^, while a study for slow-switching networks is presented in ref.^31^. Further, the compound effect of different time scales between structure dynamics and unit dynamics has been studied in ref.^32^.

The problem of controlling networked mobile oscillators has received many research interests^33–37^. However, moving-agent systems commonly have features of spatial distribution, communication constraints, and limited sensing capacity, which makes centralized control strategies generally too expensive or even infeasible to be implemented in practice. Therefore, many adaptive control strategies which require only local information, termed as decentralized strategies, have been proposed in the last decade. For instance, an adaptive strategy has been designed for the consensus of networked moving agents^38^. Further, the situation of input saturation has been taken into consideration^39^. Because of the advantages of the decentralized method, adaptive control strategies have also been extensively developed for networks with static structures^40–42^.

However, most existing adaptive control strategies are realized by continuously adjusting coupling strength between agents, here we referred as *coupling adaption strategy*. In this paper, we propose a strategy where agents may adaptively choose neighboring ones to establish temporary connections without tunning coupling strength, which we refer to as *connection adaptation strategy*. We prove that the temporary networks generated from our strategy have the same synchronization stability as that of the static networks with the same Laplacian spectrum. We further show that the resulting structure is closely related to unidirectional chains. Our study suggests that for such chains there exists a certain range of coupling strength within which the whole chain could be controlled regardless of the length of the chains. This feature further indicates that in this coupling range the networked mobile chaotic agents could be controlled regardless of the speed of the agents. Since the resulting networks generated by our strategy are always unweighted, this connection adaption strategy may be simpler to be implemented than the coupling adaptation strategies.

## Model and theory

We start from an ensemble of *N* moving agents networked on a unit planar space Γ = [0,1]^2^ (with periodic boundary conditions). Agent *i* budges with velocity ${{\bf{v}}}_{i}(t)=(v\,\cos \,{\theta }_{i},v\,\sin \,{\theta }_{i})$. The module *v* (equal for all agents) is constant, and the direction *θ*_*i*_ is randomly drawn from the interval [−*π*, *π*) with uniform probability (at each time step). The position *y*_*i*_(*t*) of agent *i* evolves, therefore, as ${y}_{i}(t+{\rm{\Delta }}t)={y}_{i}(t)+{{\bf{v}}}_{i}(t){\rm{\Delta }}t$, where Δ*t* is an integration step size.

All agents carry an identical chaotic dynamics, described by ${\dot{{\bf{x}}}}^{i}={\bf{F}}({{\bf{x}}}^{i})$, with ${{\bf{x}}}^{i}\in {{\mathbb{R}}}^{m}$, *i* = 1, 2, …, *N*, dot denoting temporal derivative, and ${\bf{F}}:{{\mathbb{R}}}^{m}\to {{\mathbb{R}}}^{m}$. Each agent builds its connections based on the state and position information that it collects from other agents located within a neighborhood of radius *r*. The interacting structure is described by the un-weighted elements of the (generically asymmetric) coupling matrix **G**(*t*) where ${g}_{ij}(t)=-1$ when agent *i* forms a directed connection with agent *j*, and *g*_*ij*_(*t*) = 0 otherwise. The diagonal elements ${g}_{ii}(t)=-{\sum }_{j\ne i}{g}_{ij}(t)$ warrant the zero-row-sum property of **G**(*t*). The dynamics of each agent is ruled by

1 $${\dot{{\bf{x}}}}^{i}={\bf{F}}({{\bf{x}}}^{i})-\sigma \sum _{j\mathrm{=1}}^{N}{g}_{ij}(t){\bf{H}}({{\bf{x}}}^{j}),$$

where *σ* is the coupling strength, and ${\bf{H}}:{{\mathbb{R}}}^{m}\to {{\mathbb{R}}}^{m}$ is here a coupling function.

**Figure 1:**
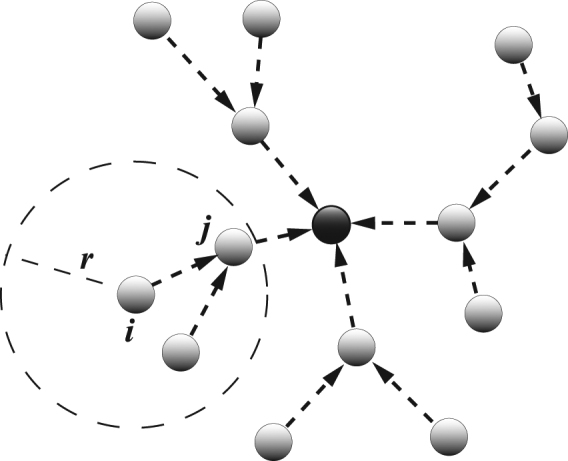
The control scheme. For clarity, the guide agent (GA) is located at the center of the square. The gray circles (dashed arrow) indicate the agents (the directed connections). Agent *i* only detects the state and position of other agents that are within the dashed circle with radius *r*. In the example, agent *i* builds a connection to agent *j* since agent *j* is the neighbor which has the smallest distance to the GA and is closer to the GA than the agent *i*. The resulting structure is a directed tree, with each agent having one out-going connection converging from the periphery to the GA.

The purpose is to steer the dynamics of all agents towards a desired solution ${{\bf{x}}}^{1}={{\bf{x}}}^{2}=\cdots ={{\bf{x}}}^{N}={{\bf{x}}}^{S}$. To this aim, we first put a unit (carrying the desired state **x** ^*s*^) at an arbitrary position in the space. Such a unit (which can be regarded actually as a virtual agent) plays the role of a reference guide for all other agents, so that we refer to it in the following as the *guide agent* (GA). The position of the GA is fixed once forever, and is known to all other agents. At each time step *t*, agent *i* chooses *one* of its neighbors (say agent *j*) to form a direct connection (so that *g*_*ij*_ = −1). The chosen neighbor is the one which satisfies two conditions: *i*) it is the nearest one (among all neighbors of agent *i* within a disk of radius *r*) to the GA (if the GA is a neighbor of agent *i*, it will therefore be chosen); *ii*) the distance between the connected agent and the GA is smaller than that between agent *i* and the GA. This latter condition ensures that all formed connections uniformly point from the periphery to the GA, and therefore the interaction structure is acyclic. Further, if no agents are found in the disk satisfying the two above conditions, agent *i* expands its interaction radius until finding a valid connection. The resulting structure is schematically sketched in Fig. 1. Note that, though the GA is located at the center of the square in Fig. 1, the position of GA can be arbitrary.

The resulting coupling matrix **G**(*t*) adaptively changes in time. After a proper relabeling of the agents [assigning the index 1 to the GA at all times, while giving smaller (larger) indices to agents closer (further) to the GA], an orthogonal transformation ***M***(*t*) can be defined, by means of which one gets a lower triangular matrix ${\bf{L}}(t)=\{{l}_{ij}(t)\}\in {{\mathbb{R}}}^{(N+\mathrm{1)}\times (N+\mathrm{1)}}$ with ${\bf{L}}(t)={{\bf{M}}}^{{\rm{T}}}(t){\bf{G}}(t){\bf{M}}(t)$ as follows,

2 $${\bf{L}}(t)=(\begin{array}{llll}0 &  &  & 0\\ \qquad \quad 1 &  & \\  &  & \ddots  & \\ {l}_{ij}=0,-1 &  &  & 1\end{array}),$$

where *l*_11_ = 0 and *l*_*ii*_ = 1(*i* ≠ 1). The values of the elements *l*_*ij*(*i*>*j*)_ [the lower part of **L**(***t***)] are either 0 or −1. Solving the related secular equation gives the spectrum of **L**(*t*) [and also of **G**(*t*)] as *λ*_1_ = 0 and ${\lambda }_{2}=\cdots {\lambda }_{N+1}=1$. The eigen-vector of *λ*_1_ corresponds to the synchronization manifold **x** ^*s*^, while the other eigen-vectors are associated to eigen-modes spanning the transverse space of the synchronization manifold. As all such eigen-modes have always the same eigenvalue, they form a unique subspace which is invariant with time. It can be proved that the condition of stable synchronization for this time-varying structure is similar to that for a static network with the same spectrum (see Methods for details on both cases of slow- and fast-varying networks).

For a time-independent coupling matrix, a necessary condition for synchronization is that the Master Stability Function (MSF) Φ(*σλ*_*i*_) be strictly negative in each transverse mode^43^. For our control scheme, the guess is therefore that the attainment of **x** ^*s*^ would occur at that value of the coupling strength *σ* for which Φ(*σ*) *< *0.

For the sake of illustration, let us now refer to the case of agents carrying the chaotic Rössler oscillator. The dynamics of each unit is therefore described by ${\dot{x}}_{1}^{i}=-({x}_{2}^{i}+{x}_{3}^{i})$, ${\dot{x}}_{2}^{i}={x}_{1}^{i}+a{x}_{2}^{i}$, ${x}_{3}^{i}=b+{x}_{3}^{i}({x}_{1}^{i}-c)$, with ${{\bf{x}}}^{i}=({x}_{1}^{i},{x}_{2}^{i},{x}_{3}^{i}{)}^{{\rm{T}}}$ and $a=b=0.2$, *c* = 7. ${\bf{H}}({\bf{x}})=({x}_{1},0,0)$, which realizes a linear coupling on the *x*_1_ variable of the agents. The dynamics is integrated with fixed integration time step Δ*t* = 0.001. Unless otherwise specified, network’s parameters are *N* = 100 and *r* = 0.1. The error function ${\delta }_{i}(t)=\frac{1}{3}(|{x}_{1}^{i}-{x}_{1}^{1}|+|{x}_{2}^{i}-{x}_{2}^{1}|+|{x}_{3}^{i}-{x}_{3}^{1}|)$ is monitored to evaluate the control performance on agent *i*. $\delta (t)=\frac{1}{N}{\sum }_{i\mathrm{=2}}^{N+1}{\delta }_{i}(t)$ (the average error of all the agents) and 〈*δ*〉 (the time averaged value of *δ*(t) over the last 10^5^ integration steps) are evaluated after a suitable transient time, to quantify the global control performance.

With such a choice of parameters (and of output function **H**), the Rössler system belongs to class III MSF^3^, i.e. Φ is negative within the range [*α*_1_, *α*_2_] (with ${\alpha }_{1}\simeq 0.2$ and ${\alpha }_{2}\simeq 4.3$). The necessary condition for stability of the synchronized solution **x** ^*s*^ is therefore:

3 $${\alpha }_{1} < \sigma  < {\alpha }_{2}\mathrm{.}$$

## Results

**Figure 2:**
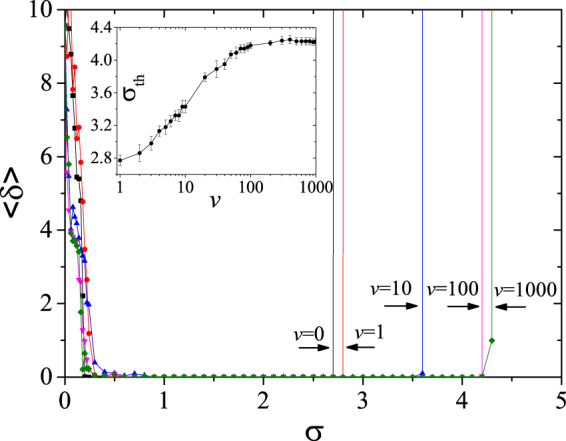
〈*δ*〉 *vs. σ* for *v* = 0,1,10,100 and 1,000. The black arrows indicate the thresholds of *σ*, denoted as *σ*_th_, above which 〈*δ*〉 diverges. The inset shows the numerically found relationship between *σ*_th_ and *v*.

Figure 2 reports 〈*δ*〉 (the control performance) *vs. σ* for various values of agents’ velocities. Remarkably [and together with the necessary condition of Eq. (3)], agents’ velocity impacts the control performance, with a range of *σ* for control which monotonically decreases with decreasing *v* (see the inset of Fig. 2, from which it is clear that only when *v* is large enough, the condition (3) is matched). A major conclusion is the existence of a range of the coupling strength (the non-vanishing range of *σ* at *v* = 0) where the system can be controlled for all agents’ velocities.

**Figure 3:**
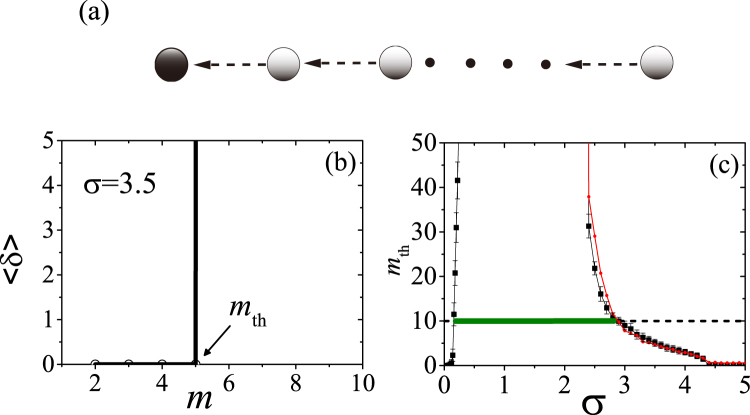
(**a**) Diagram of a unidirectional chain. Black arrows indicate directed links between the agents. As in Fig. 1, the black circle denotes the GA and the gray circles denotes the agents. (**b**) Control error 〈*δ*〉 *vs*. the length *m* of the chain, for *σ* = 3.5. A threshold *m*_th_ is observed, above which 〈*δ*〉 diverges. (**c**) *m*_th_ *vs. σ*. The green bar indicates the feasible range of *σ* for *m*_th_ = 10. The dashed line is a guide for eyes. The red curve is, instead, the result of our analysis (see text).

In order to better elucidate the control mechanism, we first concentrate on the case *v* = 0, where the structure of connections is static and can be regarded as the overlap of a group of unidirectional chains, converging towards the GA and bifurcating at some intermediate points. Since agents closer to the GA are not influenced by those further to them, the chains can be considered as independent to each other. Therefore, controlling the case *v* = 0 is tantamount to finding under which conditions unidirectional chains can be controlled to **x** ^*s*^. In the following, therefore, we focus on the performance of a typical unidirectional chain [sketched in Fig. 3(a)], for which the only structural factor is its length *m*, i.e. the number of agents to be controlled [indexed by their distance to the GA, and denoted by the gray circles in Fig. 3(a)]. Figure 3(b) reports the relation between the error function 〈*δ*〉 and *m*, at *σ* = 3.5. Remarkably, a threshold value *m*_th_ = 5 exists, such that the chain cannot be controlled for *m* > *m*_th_. Notice that *σ* = 3.5 still satisfies the necessary condition (3), which means that the distance of the agent to the GA fundamentally affects the control performance. Figure 3(c) reports the behavior of *m*_th_ as a function of *σ*. With the shape of the curve (reminiscent of the V-shape of the MSF for such coupled Rössler oscillators), *m*_th_ is negatively correlated with the MSF, and reaches 0 at the points of *α*_1_ and *α*_2_. As shown in Fig. 3(b), under a given coupling strength *σ* the feasible length of a chain for control is restricted by the threshold *m*_th_, therefore the control performance for a static network is determined by its longest chain: if the length of the longest chain, *l*_max_, is smaller (larger) than *m*_th_(*σ*) the network can (cannot) be controlled. We verified that *l*_max_ ~ 10 (for our network with *v* = 0 and *r* = 0.1), and the corresponding range of *σ* satisfying ${m}_{{\rm{th}}}(\sigma ) > 10$ is marked by the horizontal green bar in Fig. 3(c). The range of coupling spanned by the green bar is well consistent with that for the case of *v* = 0 in Fig. 2. We have also compared *l*_max_ with *m*_th_ for different radius *r*, and good consistency appears in all of them (see Supplemental Information).

**Figure 4:**
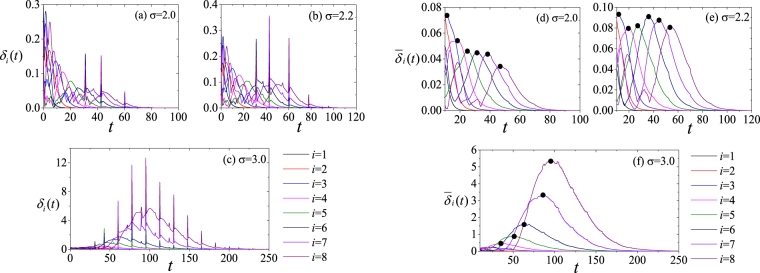
(Left panels) Time evolution of *δ*_*i*_(*t*) (*i* = 1, …,8) for *σ* = 2.0 (**a**), 2.2 (**b**), and 3.0 (**c**); (Right panels) Time evolution of ${\bar{\delta }}_{i}(t)$ (*i* = 1, …, 8) for *σ* = 2.0 (**d**), 2.2 (**e**), and 3.0 (**f**). Distinct humps appear before ${\bar{\delta }}_{i}(t)$ vanishes. Black dots mark the peak of the humps of ${\bar{\delta }}_{i}(t)$ for a guide to the eyes.

Furthermore, it is seen in Fig. 3(c) that *m*_th_(*σ*) diverges when *σ* is in the range [0.6, 2.2] (we have verified this divergence with extensive simulations where the the length of a 1D chain is increased up to 1,000, and still synchronization occurs in that range). In order to gather more detailed information, we monitor the time evolution of *δ*_*i*_(*t*), and find that *δ*_*i*_(*t*) features intermittent spikes (see Fig. 4(a–c)), which is actually caused by sudden surges in the *x*_3_ variable of the Rössler oscillator. In order to capture the main profile of the evolution, one can then use a smoothing process to remove the spikes. More precisely, a Smooth function ${\bar{\delta }}_{i}(t)=\frac{1}{2\tau }{\int }_{t-\tau }^{t+\tau }{\delta }_{i}(t^{\prime} )dt^{\prime}$ is defined. As the typical time interval spanned by the spikes is about 2 = 2 × 10^3^Δ*t*, when *τ* is larger than 2 they are eliminated. Figure 4(d–f) show the resulting profile after applying the smoothing function, for *τ* = 10. One observes that, before vanishing, ${\bar{\delta }}_{i}(t)$ features a characteristic hump, whose height ${\bar{\delta }}_{i}^{h}$ (indicated by the black dots), changes with the index *i*. For instance, for *σ* = 3, ${\bar{\delta }}_{i}^{h}$ increases with increasing *i*, leading eventually to a divergence of ${\bar{\delta }}_{i}$. For *σ* = 2, instead, ${\bar{\delta }}_{i}^{h}$ does not increase with *i*, suggesting that the whole chain can be controlled even when its length is very long. These humps can be regarded as transient fluctuations, that can be delivered from one agent to the next. For some coupling strength *σ*, these fluctuations will be scaled up during the delivery. To accurately describe the tendency of ${\bar{\delta }}_{i}^{h}$ evolving with *i*, one then defines the ratio between the humps of two adjacent agents ${\bar{\delta }}_{i}^{h}$ as ${\gamma }_{i}(\sigma )={\bar{\delta }}_{i+1}^{h}/{\bar{\delta }}_{i}^{h}$, and the average of such ratios on the chain as $\gamma (\sigma )=\frac{1}{{m}_{{\rm{th}}}\,-\,1}{\sum }_{i\mathrm{=1}}^{{m}_{{\rm{th}}}-1}{\gamma }_{i}(\sigma )$, which then measures the converging (or diverging) tendency of the whole chain.

**Figure 5:**
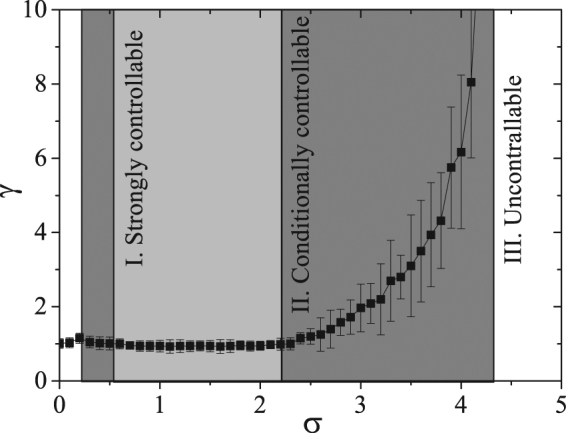
*γ* (see text for definition) as a function of *σ*. The entire range of *σ* can be separated into three regions: I. Strongly controllable region, where ${\rm{\Phi }}(\sigma \mathrm{) < 0}$ and $\gamma \lesssim 1$ (Light gray); II. Conditionally controllable region, where ${\rm{\Phi }}(\sigma ) < 0$ and *γ* > 1 (Dark gray); III. Uncontrollable region, where Φ(*σ*) > 0 (White).

*γ*(*σ*) is reported in Fig. 5. It is seen that, when 0.6 < *σ* < 2.2, $\gamma \simeq 1$, and therefore ${\bar{\delta }}_{i}^{h}$ does not scale up with increasing *i*. When instead *σ* is out of such a range, ${\bar{\delta }}_{i}^{h}$ is amplified with the increase of the index *i*, and eventually exceeds a certain domain, leading to divergence of the state. By calling the size of the domain as *U*, then one has ${\bar{\delta }}_{1}^{h}{\langle \gamma (\sigma )\rangle }^{{m}_{{\rm{th}}}^{^{\prime} }-1}=U$, where ${\bar{\delta }}_{1}^{h}$ is the height of the hump of the first agent (i.e. the initial value), and ${m^{\prime} }_{{\rm{th}}}$ denotes the number of amplifications needed to reach *U*. As a consequence, $m{{\rm{^{\prime} }}}_{{\rm{th}}}=ln(U/{\bar{\delta }}_{1})/ln\langle \gamma (\sigma )\rangle +1$. The value of $U/{\bar{\delta }}_{1}\mathrm{=200}$ is the result of a fit, and one can further input the dependencies of $\gamma (\sigma )$, so as to obtain a prediction for ${m^{\prime} }_{{\rm{th}}}$. ${m^{\prime} }_{{\rm{th}}}$ as a function of *σ* is reported as a red dashed curve in Fig. 3(c). One can easily see how significantly the two curves for ${m^{\prime} }_{{\rm{th}}}$ and *m*_th_ are matching, pointing to the fact that the parameter *γ* represents actually a proper indicator to predict the tendency of ${\bar{\delta }}_{i}^{h}$.

As a conclusion of all the above reasoning, our results suggest that one can partition all the range of *σ* into three distinct regions (reported in Fig. 5): Region I, characterized by ${\rm{\Phi }}(\sigma ) < 0$ and $\gamma \lesssim 1$. In this region synchronization occurs regardless of the length of the chain, so that we can safely call this region as *Strongly controllable region* (SCR); Region II, where ${\rm{\Phi }}(\sigma ) < 0$ but *γ* > 1. Here, though the necessary condition of the MSF is satisfied, synchronization is restricted to a finite chain length, so that the region is called as *Conditionally controllable region*; Region III, where Φ(*σ*) > 0. Here the necessary condition of a negative MSF is not satisfied. Therefore, synchronization is intrinsically unstable, and we call this region as the *Uncontrollable region*.

Finally, we briefly elaborate on the case where *v* ≠ 0. There, network’s chains are continuously broken and regrouped, with such a process occurring more often at higher agents’ velocities. When a chain is broken, the fluctuation delivery process is interrupted and current fluctuations may be defused on the newly formed chain. Therefore, increasing the velocity *v* is equivalent to reducing *l*_max_. For instance, at *v* = 10, the network can be controlled when *σ* < 3.6 (see Fig. 2), which indicates that chains with lengths longer than 5 (compare with data and discussion on Fig. 3) cannot remain unchanged long enough (under this speed) to induce an effective divergence of the state variables. On the other hand, when *v* is small, long chains are more easily to remain unmodified, and control therefore fails.

## Conclusion

In this paper, we have proposed an adaptive strategy for controlling networked mobile chaotic oscillators with local information. The key idea of this strategy is to allow each agent to select one of its neighbors to form up a connection between them. The resulting temporary structures generated by this strategy is a set of directed trees and their Laplacian spectrum are always kept the same with identity eigenvalues for all the transversal modes. We have proved that such a time-varying structure has the same synchronization stability as that of the static network with the same Laplacian spectrum. Thus, the networks may be controlled without changing coupling strength. We have further shown that the temporary structure generated by the proposed strategy is closely related to a unidirectional chain. By investing this featured structure, we observe a specific region of coupling strength, called *strongly controllable region*, within which the whole chain can be controlled regardless of its length. This feature is of value for our strategy, since in this region the whole networks can be controlled for arbitrary moving speed of the oscillators.

Compared to the dominant adaptive control scheme for related mobile systems where the coupling strength is adjusted continuously, our strategy may be regarded as a favorable simplification where the coupling ratio only needs to be set as either 0 or 1 regardless of the states of the oscillators, hence significantly reduced the efforts for implementation. We expect our work may inspire more researches in developing simple and efficient decentralized strategies for the control of networked mobile systems. Though in this work the results are presented on a simple model, the proposed scheme can be extended to more realistic situations, e.g., where there exist noises, errors or non-negligible delay in communications between agents and systems are of complicated spacial structures, etc. Such extensions will be our future research interest.

## Methods

### The necessary condition for control

The linearized equation of the system deviating around the solution ${{\bf{x}}}^{S}\equiv {{\bf{x}}}^{1}={{\bf{x}}}^{2}=\mathrm{....}={{\bf{x}}}^{N+1}$ can be written as

4 $$\delta \dot{{\bf{x}}}=[{{\bf{I}}}_{N+1}\otimes D{\bf{F}}-\sigma {\bf{G}}\otimes D{\bf{H}}]\delta {\bf{x}},$$

where $\delta \dot{{\bf{x}}}={(\delta {\dot{{\bf{x}}}}^{1},\cdots ,\delta {\dot{{\bf{x}}}}^{N+1})}^{{\rm{T}}}$, **I**_*N*+1_ is the identity matrix of order *N* + 1, and *D* **F** and *D* **H** are the Jacobian functions of **F** and **H**, respectively. This equation is in accordance to Eq. (1), and we here label the GA as agent 1 and the other *N* agents with indices from 2 to *N* + 1. At time *t*, the Laplacian **G** is decomposed as ${\bf{G}}={\sum }_{i\mathrm{=1}}^{N}{\lambda }_{i}{{\bf{u}}}_{i}{{\bf{u}}}_{i}^{{\rm{T}}}$, where the spectrum {*λ*_*i*_} is such that *λ*_1_ = 0 and ${\lambda }_{2}=\cdots ={\lambda }_{N+1}=1$. The eigen-vectors $\{{{\bf{u}}}_{i}={({u}_{i1},\cdots ,{u}_{i,N+1})}^{{\rm{T}}}\}$ form an ortho-normal basis, with ${{\bf{u}}}_{1}=\frac{1}{\sqrt{N}}{(1,\cdots ,1)}^{{\rm{T}}}$ corresponding to the synchronization manifold **x** ^*s*^. Taking ${{\bf{I}}}_{N+1}={\sum }_{i\mathrm{=1}}^{N+1}{{\bf{u}}}_{i}{{\bf{u}}}_{i}^{{\rm{T}}}$, one gets

5 $$\delta \dot{{\bf{x}}}=\sum _{i\mathrm{=1}}^{N+1}[{{\bf{u}}}_{i}{{\bf{u}}}_{i}^{{\rm{T}}}\otimes (D{\bf{F}}+\sigma {\lambda }_{i}D{\bf{H}})]\delta {\bf{x}}{\boldsymbol{.}}$$

Setting *δ* **x**(*t*) = **Q** *δ* **y**(*t*) with ${\bf{Q}}=({{\bf{u}}}_{1},\ldots ,{{\bf{u}}}_{N+1})\otimes {{\bf{I}}}_{m}$, where **I**_*m*_ is the identity matrix of order *m* (*m* being the number of degrees of freedom of the dynamics carried by each agent), Eq. (5) becomes

6 $$\delta \dot{{\bf{y}}}={{\bf{Q}}}^{-1}\sum _{i\mathrm{=1}}^{N+1}[{{\bf{u}}}_{i}{{\bf{u}}}_{i}^{{\rm{T}}}\otimes (D{\bf{F}}+\sigma {\lambda }_{i}D{\bf{H}})]{\bf{Q}}\delta {\bf{y}}=\sum _{i\mathrm{=1}}^{N+1}[{{\bf{e}}}_{i}{{\bf{e}}}_{i}^{{\rm{T}}}\otimes (D{\bf{F}}+\sigma {\lambda }_{i}D{\bf{H}})]\delta {\bf{y}},$$

where **e**_*i*_ is a unit vector whose *i*-th element is 1 and 0 otherwise. The matrix in Eq. (6) is block diagonal with *m* × *m* blocks. Taking *δ* **y** as *δ* **y** = (*δ* **y** ^1^, …, *δ* **y** ^*N*^)^T^ with $\delta {{\bf{y}}}^{i}\in {{\mathbb{R}}}^{m}$, one has

7 $$\delta {\dot{{\bf{y}}}}^{i}=(D{\bf{F}}+\sigma {\lambda }_{i}D{\bf{H}})\delta {{\bf{y}}}^{i}\mathrm{.}$$

Since *δ* **y** = **Q** ^T^ *δ* **x** (and noting that *λ*_*i*_ = 1 for *i* ≥ 2), Eq. (7) gives

8 $$\sum _{j\mathrm{=2}}^{N+1}{u}_{ij}\delta {\dot{{\bf{x}}}}^{j}=(D{\bf{F}}+\sigma D{\bf{H}})\sum _{j\mathrm{=2}}^{N+1}{u}_{ij}\delta {{\bf{x}}}^{j},\,{\rm{for}}\,i\ge 2.$$

Since here {*u*_*ij*(*i*,*j*≥2)_} can be arbitrary set as long as they form an orthonormal basis of eigen-vectors, the solution for *δ* **x** ^*j*^ can only be

9 $$\delta {\dot{{\bf{x}}}}^{j}=(D{\bf{F}}+\sigma D{\bf{H}})\delta {{\bf{x}}}^{j},\,{\rm{for}}\,j\ge 2.$$

Therefore, when the spectrum of **G**(*t*) satisfies *λ*_1_ = 0 and *λ*_2_ = …*λ*_*N*+1_ = 1, the deviation around **x** ^*s*^ of all the agents (except for the GA) shall evolves uniformly following Eq. (9).

The above argument reveals how *δ* **x** evolves during the time window where the structure is fixed. Now, let us discuss what happens, instead, when the structure switches from one topology to another due to the movement of the agents in the square plane. When this happens, say at time $t^{\prime}$, then *δ* **x**(*t*′) (the final solution of the deviation during the presence of the previous structure of connections) now becomes the initial condition for the evolution of the deviation in the presence of the new structure. It is important to remark that the eigen-vectors of the new structure may be different from the old ones (though the eigenvalues’ spectra are the same). Specifically, we suppose the new structure could be decomposed as ${\bf{G}}={\sum }_{i\mathrm{=1}}^{N}{\lambda }_{i}{{\bf{v}}}_{i}{{\bf{v}}}_{i}^{{\rm{T}}}$ with eigenvectors ${{\bf{v}}}_{i}=({v}_{i1},\cdots ,{v}_{i,N+1}{)}^{{\rm{T}}}$. Similarly, setting $\delta {\bf{x}}(t^{\prime} )={\bf{R}}\delta {\bf{z}}(t^{\prime} )$ with ${\bf{R}}=({{\bf{v}}}_{1},\cdots ,{{\bf{v}}}_{N+1})\otimes {{\bf{I}}}_{m}$, one obtains

10 $$\delta {\dot{{\bf{z}}}}^{i}=(D{\bf{F}}+\sigma {\lambda }_{i}D{\bf{H}})\delta {{\bf{z}}}^{i}\mathrm{.}$$

Owing to the difference between **Q** and **R** (i.e. the difference of the eigenvectors), *δ* **y** ^*i*^(*t*′) and *δ* **z** ^*i*^(*t*′) could be different. That is to say that the change of eigenvectors may lead to a change of the deviations of eigen-transversal modes for different structures, so that *δ* **y** ^*i*^(*t*′) changes to *δ* **z** ^*i*^(*t*′).

However, since both Eqs (7) and (10) bring about Eq. (9), and *δ* **x**(*t*′) is maintained at the moment at which the structure switches, we conclude that Eq. (9) is valid at all times during the structure switching process. Therefore, since in our method the spectrum of the temporal Laplacians always satisfies the condition, we conclude that the criteria for stability of the solution where all agents are synchronized with the GA are equivalent to those for the same solution in a static network featuring the same eigenvalues’ spectrum.

## Electronic supplementary material

Supplementary Information
