# Supplementary material for: Connection adaption for control of networked mobile chaotic agents
Source: Sci Rep. 2017 Nov 22;7:16069. doi: 10.1038/s41598-017-16235-2 (PMC5700208; doi:10.1038/s41598-017-16235-2)
Supplement: Supplementary file 1 — Supplementary Information [file 41598_2017_16235_MOESM1_ESM.pdf]

Supplemental Information:
Connection adaption for control of networked mobile chaotic agents

Jie Zhou,[1] Yong Zou,[1] Shuguang Guan,[1] Zonghua Liu,[1] Gaoxi Xiao,[2,3] and S. Boccaletti[4,5]

[1] *Department of Physics, East China Normal University, Shanghai 200241, China*
[2] *School of Electrical and Electronic Engineering, Nanyang Technological University 639798, Singapore*
[3] *Complexity Institute, Nanyang Technological University 639798, Singapore*
[4] *CNR-Institute of Complex Systems, Via Madonna del Piano, 10, 50019 Sesto Fiorentino, Florence, Italy*
[5] *The Embassy of Italy in Tel Aviv, 25 Hamered street, 68125 Tel Aviv, Israel*

THE IMPACT OF CONTACT RADIUS

Figure 1(a) shows the length of largest chain, $l_{\max}$, as a function of the contact radius r for the case of $v = 0$. We observe the inverse correlation between them, and the inset in Fig. 1(a) highlights that the curve $l_{\max}$ *vs.* $1/r$ displays indeed a linear fit, with a slope around 1. As pointed out in the main text, for a certain coupling strength σ, when $l_{\max}$ does not exceed $m_{\rm th}(\sigma)$ the whole network can be controlled. As increasing r causes a decrease of $l_{\max}$, an enlargement of the feasible range of σ for control should also be determined. The latter is confirmed in Fig. 1(b), where $\langle\delta\rangle$ is reported *vs.* σ, for different r values. The striking similarity of the trends reported in Fig. 1(b) here and in Fig. 2 in the main text suggests that the effect of increasing the velocity v is actually equivalent to that of reducing the length of the longest chain $l_{\max}$. Figure 1(b) suggests the existence of an upper bound of σ, denoted as $\sigma_{\rm th}$, above which $\langle\delta\rangle$ diverges, for different r. The relation between r and $\sigma_{\rm th}$ is presented in Fig. 1(c). Combining Figs. 1(a) and (c), one obtains the relation between $\sigma_{\rm th}$ and $l_{\max}$ as shown in Fig. 1(d), where the function of $m_{\rm th}(\sigma)$ is also plotted for comparison. We can see that $l_{\max}(\sigma_{\rm th})$ matches well with $m_{\rm th}(\sigma)$, conforming to our conclusion in the main text that the control performance for a static network is determined by the longest chain.

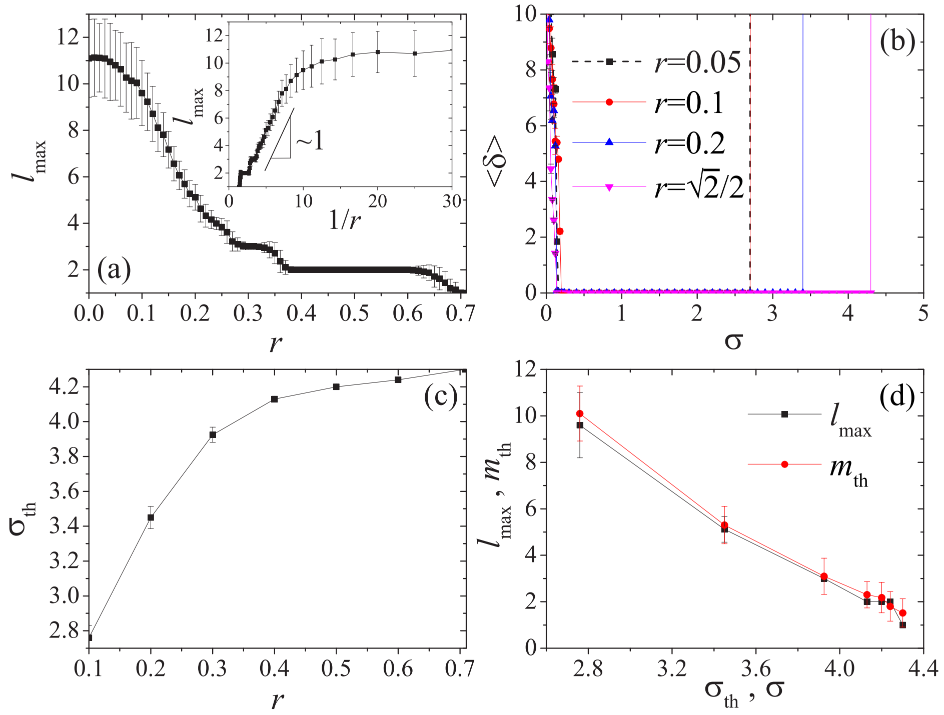

FIG. 1: (Color online). (a) Length of the longest chain $l_{\max}$ as a function of r. $l_{\max}$ is around 11 at $r < 0.1$. Inset: $l_{\max}$ *vs.* $1/r$. (b) $\langle\delta\rangle$ *vs.* σ for $r = 0.05, 0.1, 0.2$, and $\sqrt{2}/2$, and for $v = 0$. Note that when $r = \sqrt{2}/2$ all the agents connect uniformly to the GA. (c) The relationship between r and the threshold $\sigma_{\rm th}$, above which $\langle\delta\rangle$ diverges. (d) The relationship between $l_{\max}$ and $\sigma_{\rm th}$, which is produced with the help of panels (a) and (c), and the relation between $m_{\rm th}$ and σ, reported already in Fig. 3(c) in the main text. Other parameters are the same as those in the main text.
